# LC-Orbitrap-HRMS method for analysis of traces of triacylglycerols featuring furan fatty acids

**DOI:** 10.1007/s00216-022-04480-y

**Published:** 2022-12-20

**Authors:** Nina Wiedmaier-Czerny, Walter Vetter

**Affiliations:** Institute of Food Chemistry, Department of Food Chemistry (170b), University of Hohenheim, 70593 Stuttgart, Germany

**Keywords:** Antioxidant, Furan fatty acid, Triacylglycerol, Lipidomic, LC-Orbitrap-HRMS

## Abstract

Furan fatty acids (FuFAs) are valuable antioxidants that are highly relevant for the protection of polyunsaturated fatty acids (PUFAs) in biological systems and food. Despite their low contributions to the total fatty acids, their widespread occurrence has been documented in food and biological samples. Like other fatty acids, FuFAs are also stored esterified, e.g., in triacylglycerols. However, FuFA-containing triacylglycerols had not been detected in lipidomics analyses. Here, we present a screening method that allows for the identification of traces of FuFA-containing triacylglycerols (TAGs) utilizing LC-Orbitrap-HRMS. Initially developed with the help of purposefully synthesized FuFA-containing TAGs, the screening method was successfully applied to the analysis of two fish oil samples and one mushroom extract sample. Several FuFA-containing TAGs could be identified by direct analysis using the method and database developed in this study.

**Graphical Abstract:**

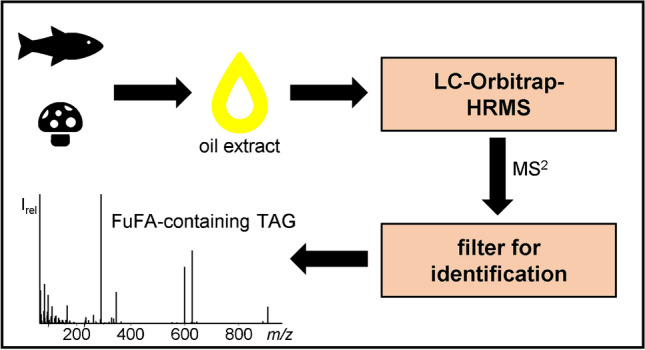

**Supplementary Information:**

The online version contains supplementary material available at 10.1007/s00216-022-04480-y.

## Introduction

**Fig. 1:**
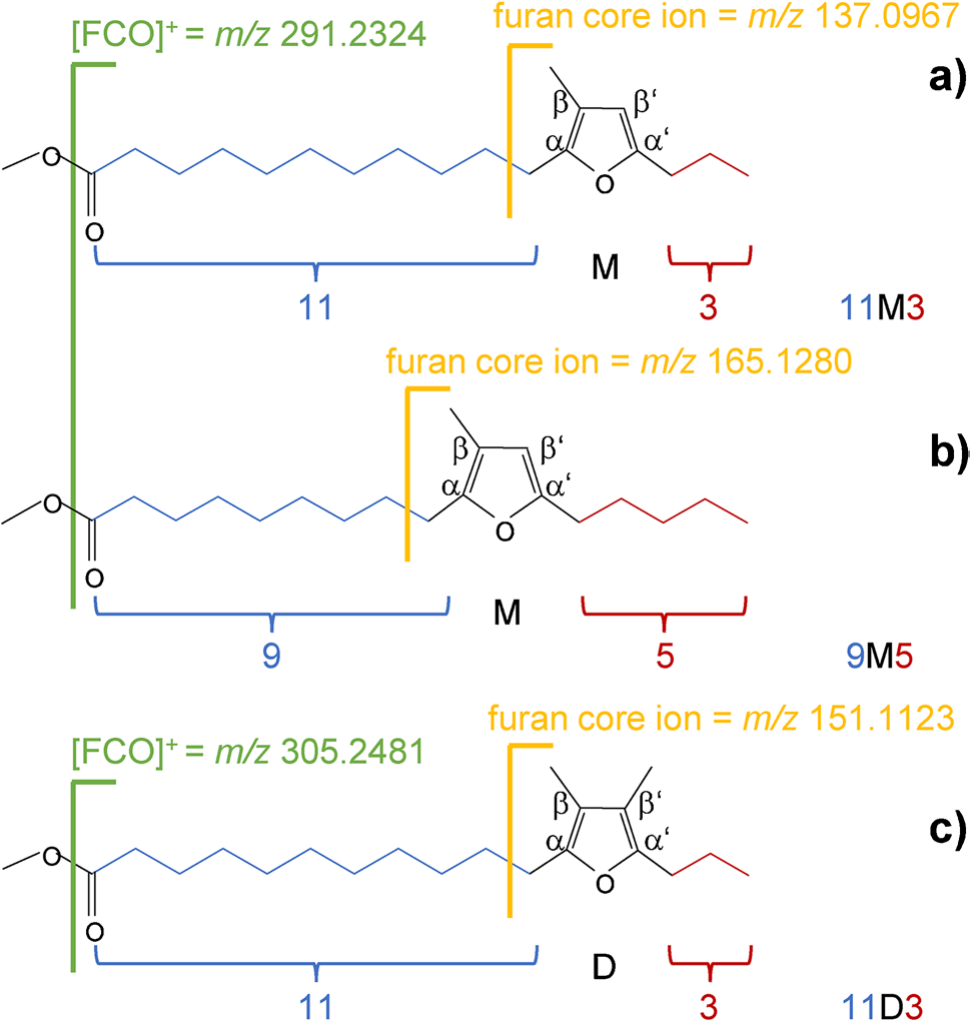
Chemical structures of (**a**) 11-(3-methyl-5-propylfuran-2-yl)-undecanoic acid (11M3), (**b**) 9-(3-methyl-5-pentylfuran-2-yl)-nonanoic acid (9M5), and (**c**) 11-(3,4-dimethyl-5-propylfuran-2-yl)-undecanoic acid with illustrations for the short terms (below) and characteristic fragment ions in MS^2^ spectra of LC-Orbitrap-HRMS measurements

Furan fatty acids (FuFAs) are powerful radical scavengers that can effectively prevent the oxidation of polyunsaturated fatty acids (PUFAs) and other susceptible molecules [1–3]. Arguably, FuFAs thus belong to the most valuable fatty acids in living organisms and the human diet. For instance, some beneficial health effects of fish consumption currently linked to PUFAs were proposed to be caused by FuFAs [1]. Structurally, the most relevant FuFAs feature a furan moiety with an odd-numbered carboxyalkyl chain of mainly nine or eleven carbon atoms attached in α-position and an alkyl chain of three or five carbon atoms in α′-position (Fig. 1). In addition, either one or two methyl groups are located in β- and β′-position [1, 4, 5]. Instead of the complex chemical names (e.g., 9-(3-methyl-5-pentylfuran-2-yl)-nonanoic acid, Fig. 1), FuFAs can be presented by number-letter-number short forms which give direct structural information (for this example, 9M5) [4]. Namely, the first and the second number denote the number of carbon atoms of the carboxyalkyl and the alkyl chain, respectively, while the central letter indicates the methylation degree on the furan moiety (“M” = monomethyl-substituted in β-position, “D” = dimethyl-substituted in β,β′-positions) [4] (Table 1). For better readability in TAGs, numbers in these short forms (e.g., 9D5) will be presented in superscript and subscript style (e.g., ^9^D_5_) [6].

**Table 1:** Short forms, building blocks, and chemical names of the most relevant furan fatty acids (FuFAs)

Short form [4]	Short form for TAGs [6]	Length of the carboxy alkyl chain	Backbone	Length of the alkyl chain	Chemical name	No. of carbons
9M3	^9^M_3_	9	ß-Methyl-furan	3	9-(3-Methyl-5-propylfuran-2-yl)-nonanoic acid	17
9D3	^9^D_3_	9	ß,ß′-Dimethyl-furan	3	9-(3,4-Dimethyl-5-propylfuran-2-yl)-nonanoic acid	18
9M5*	^9^M_5_	9	ß-Methyl-furan	5	9-(3-Methyl-5-pentylfuran-2-yl)-nonanoic acid	19
11M3	^11^M_3_	11	ß-Methyl-furan	3	11-(3-Methyl-5-propylfuran-2-yl)-undecanoic acid	19
9D5*	^9^D_5_	9	ß,ß′-Dimethyl-furan	5	9-(3,4-Dimethyl-5-pentylfuran-2-yl)-nonanoic acid	20
11D3*	^11^D_3_	11	ß, ß′-Dimethyl-furan	3	11-(3,4-Dimethyl-5-propylfuran-2-yl)-undecanoic acid	20
11M5	^11^M_5_	11	ß-Methyl-furan	5	11-(3-Methyl-5-pentylfuran-2-yl)-undecanoic acid	21
11D5*	^11^D_5_	11	ß,ß′-Dimethyl-furan	5	11-(3,4-Dimethyl-5-pentylfuran-2-yl)-undecanoic acid	22

^*^The most important FuFAs in biological samples and food are marked with an asterisk. In addition to the eight mentioned FuFAs, 7D5 (7-(3,4-dimethyl-5-pentylfuran-2-yl)-heptanoic acid, with 18 carbon atoms) was present in fish oil II analyzed in this study

Similar to other antioxidants such as tocopherols, FuFA levels in food are considerably low (typically < 1% of the lipids) [7, 8]. Like other fatty acids, FuFAs are almost exclusively found esterified in different lipid classes. However, the structural similarity with conventional fatty acids makes the analysis of FuFAs more challenging [9] than, e.g., of tocopherols which can be easily separated from the bulk of fatty acids. Typical analysis protocols for FuFA determinations include lipid extraction followed by saponification and/or transmethylation followed by GC/MS or LC/MS determination [5, 7, 9–11]. Almost exclusively, an enrichment step by silver ion chromatography has to be carried out prior to the quantification [12]. On the one hand, all information on the presence of FuFAs in particular lipid classes is lost after these sample processing steps. On the other hand, existing methods of FuFA enrichment cannot be applied to intact lipids such as triacylglycerols (TAGs) which are characterized by a huge plethora of structural variants [13, 14].

In lipidomics, TAGs are usually analyzed by high-performance liquid chromatography in combination with electrospray tandem mass spectrometry (LC-ESI-MS^2^) [15–17]. Regardless of immense progress in LC/MS instrumentation, contemporary analysis is still hampered by the wide concentration range of individual TAGs. As a consequence, very low abundant fatty acids such as FuFAs are usually overlooked although they could be highly bioactive. Namely, the direct measurement of FuFA-containing TAGs has not been reported, yet. However, the inclusion of FuFA-containing TAGs in lipidomic studies seems to be an important matter given the high relevance of FuFAs for the prevention of lipid oxidation.

Here, we provide a method for the direct analysis of FuFA-containing TAGs in food samples by LC-Orbitrap-HRMS. Initial measurements of purposefully synthesized FuFA-containing TAGs [6] enabled us to select FuFA-specific *m*/*z* values. While the direct identification of FuFA-containing TAGs in first-dimension mass spectra (MS^1^) via the exact mass was found to be equivocal, the selection of diagnostic *m*/*z* values in second-dimension mass spectra (MS^2^) enabled their detection in two relevant sample matrices and created a database for the direct determination of FuFA-containing TAGs in food and biological samples.

## Materials and methods

### Solvents and chemicals

Methanol (99.9%), water and acetonitrile (99.9%) (all HiPerSolv CHROMANORM for LC/MS) were bought from VWR (Radnor, PA, USA). Ammonium formiate (ultra plus LC/MS grade) was from Fluka (Steinheim, Germany). *Iso*-propanol (LC/MS grade) was purchased from Biosolve (Valkenswaard, Netherland). Ethanol (> 99.9%), cyclohexane (99.5%), and *n*-hexane (> 95%) were ordered from TH. Geyer (Renningen, Germany). Ethyl acetate was bought from Sigma-Aldrich (Steinheim, Germany). The FuFA-containing TAG standards were synthesized according to Wiedmaier-Czerny et al. [6]. Fish oil I was a concentrate of ω-3 fatty acids which was rich in 5,8,11,14,17-eicosapentaenoic acid (EPA) and 4,7,10,13,16,19-docosahexaenoic acid (DHA) (Table 2) and mixed tocopherols produced by BASF (Ludwigshafen am Rhein, Germany) and was used in another study before from Masuchi Buscato et al. [18]. They analyzed FuFAs as methyl esters and found 11-(3,4-dimethyl-5-propylfuran-2-yl)-undecanoic acid (11D3) (450 mg/100 g fat), 11-(3,4-dimethyl-5-pentylfuran-2-yl)-undecanoic acid (11D5) (310 mg/100 g fat), and 9M5 (270 mg/100 g fat) as major FuFAs. Fish oil II was a liver extract of a fresh gilthead from conventional aquaculture (intensive farming) in Greece. The mushroom used was an organic king oyster mushroom (*Pleurotus eryngii*) from the supermarket. After the mushrooms were frozen, they were lyophilized in a LYOVAC GT 2 system (Leybold-Heraeus, Hürth, Germany) at 0.1 mbar. The freeze-dried mushrooms were then ground with a mill.

**Table 2:** Abbreviation list of the used fatty acids with their trivial name and their short form for TAGs

Fatty acid	Trivial name	Short form (*[30])	Short form for TAGs [31]
16:0	Palmitic acid	PA*	P
16:1Δ9	Palmitoleic acid	PnA	Pn
18:0	Stearic acid	SA*	S
18:1Δ9	Oleic acid	OA*	O
18:2Δ9,12	Linoleic acid	LA*	L
18:3Δ9,12,15	α-Linolenic acid	ALA*	Ln
20:5Δ5,8,11,14,17	Eicosapentaenoic acid	EPA	Ep
22:5Δ7,10,13,16,19	Docosapentaenoic acid	DPA	Dp
22:6Δ4,7,10,13,16,19	Docosahexaenoic acid	DHA	Dh

### Lipid extraction

About 400 mg freeze-dried king oyster mushroom powder was extracted twice with 4 mL cyclohexane/ethyl acetate (46:54, *w*/*w*) via ultrasonication (5 min) followed by centrifugation (8 min). The whole procedure was repeated in the same way with 4 mL *iso*-propanol/*n*-hexane (1:4, *v/v*). The combined supernatant of the four extractions was evaporated to dryness in a pre-weighed tube by means of a gentle stream of nitrogen. The weighed residue (~ 8.8 mg) was dissolved in 1 mL ethanol. After membrane filtration, the sample was measured by LC/MS.

For the fish oil II, a gilthead liver was freeze-dried and the oil was extracted by accelerated solvent extraction (ASE) with a Dionex ASE 350 (Thermo Scientific, Waltham, MA, USA) instrument using the instrumental parameters of Weichbrodt et al. [19]. The used solvent system was an azeotropic mixture of cyclohexane/ethyl acetate (46/54, *w*/*w*). The extract was reduced and made up to a total volume of 4 mL. An aliquot (~ 10 mg) was taken from this, the solvent removed in a heating block maintained at 40 °C with a gentle stream of nitrogen and dissolved in 1 mL ethanol.

### High-performance liquid chromatography with mass spectrometry (LC-Orbitrap-HRMS)

Samples were measured on a HPLC 1290 (Agilent, Waldbronn, Germany) instrument interfaced with a Q Exactive Plus high-resolution mass spectrometer (Thermo Scientific, Waltham, MA, US). Separations were performed with a 2.1-mm-long, 150-mm-i.d., and 1.7-µm-particle size ACQUITY UPLC CSH C18 column (Waters, Milford, MA, USA). The column temperature was set to 60 °C and 5 µL of the sample was injected. Eluent A (acetonitrile/water (6:4, *v*/*v*) with 5 mM ammonium formiate) and eluent B (*iso*-propanol/acetonitrile (9:1, *v*/*v*) with 5 mM ammonium formiate) additionally featured 0.2% formic acid. The flow rate of 0.325 mL/min was selected because it performed best during testing of 0.320–0.340 mL/min in steps of 0.005 mL/min. A gradient program from 55 to 40% A within 7 min, then 40–18% A within 21 min, was used. Finally, A decreased to 1% during 1 min, which was held for 5.90 min. After 34.90 min, the run was finished. The pump needed a further 0.10 min to get the start conditions, and these were held for 5 min. The mass spectra were acquired using electrospray ionization (ESI) in positive mode. The scan range of MS^1^ covered *m*/*z* 100–1200 with a resolution (FMHW) of 70,000. MS^2^ covered *m*/*z* 50–2000.

### Identification of triacylglycerols containing FuFAs

For identification of TAGs, characteristic fragment ions are available [20–23]. The molecular ion [M]^+^ and the high abundant [M-RCOO]^+^ are of great importance. [M-RCOO]^+^ results from the release of an entire acyl group from the molecule [24, 25]. TAGs with three identical fatty acids show only one [M-RCOO]^+^ ion (e.g., *m*/*z* 551.5 in the case of tripalmitin (PPP)). TAGs with two or three different fatty acids form [M-RCOO]^+^ ions with different *m*/*z* values such as *m*/*z* 551.5 and *m*/*z* 579.5 in the case of 1,2-dipalmitoyl-3-stearoylglycerol (PPS) [20, 24]. TAGs with three different fatty acids, all three combinations of fragmentation ions, are built, in particular [AB]^+^, [AC]^+^, [CB]^+^, for a TAG with the structure ABC. The fragment ion with lowest abundance, [AC]^+^, corresponds to the loss of the fatty acid from the *sn*-2 position [17]. But often, it is not clear in which order the fatty acids are arranged on the glycerol backbone. It is assumed that isomers co-elute due to the same mass. For the sake of simplicity, a scheme was drawn up in which fatty acids were listed in a specific order. Namely, FuFAs were always assigned to the *sn-3* position, while conventional fatty acids were arranged in ascending order of molecular weight. Note that this must not necessarily agree with the real order in the corresponding TAG.

In addition, the acyl ions [RCO]^+^ are further relevant peaks in the LC/MS spectrum. For instance, *m*/*z* 267.2 ([C_18_H_35_O]^+^) and *m*/*z* 239.2 ([C_16_H_31_O]^+^) indicate the presence of stearic acid (18:0, S) and palmitic acid (16:0, P) (Table 2) in a given TAG. Further characteristic fragment ions show a positive mass difference compared to the acyl ion (namely, + 74 u and + 128 u). These and other less prominent peaks are indicative of TAGs [24].

Beyond that, the characteristic and abundant GC/MS fragment ions of FuFA-ME (e.g., *m*/*z* 165 (base peak)) are also present in the LC/MS spectra of FuFA-containing TAGs (FuFA-TAGs) [26].

## Results and discussion

### Structural and mass spectrometric features of FuFA-containing TAGs

**Fig. 2:**
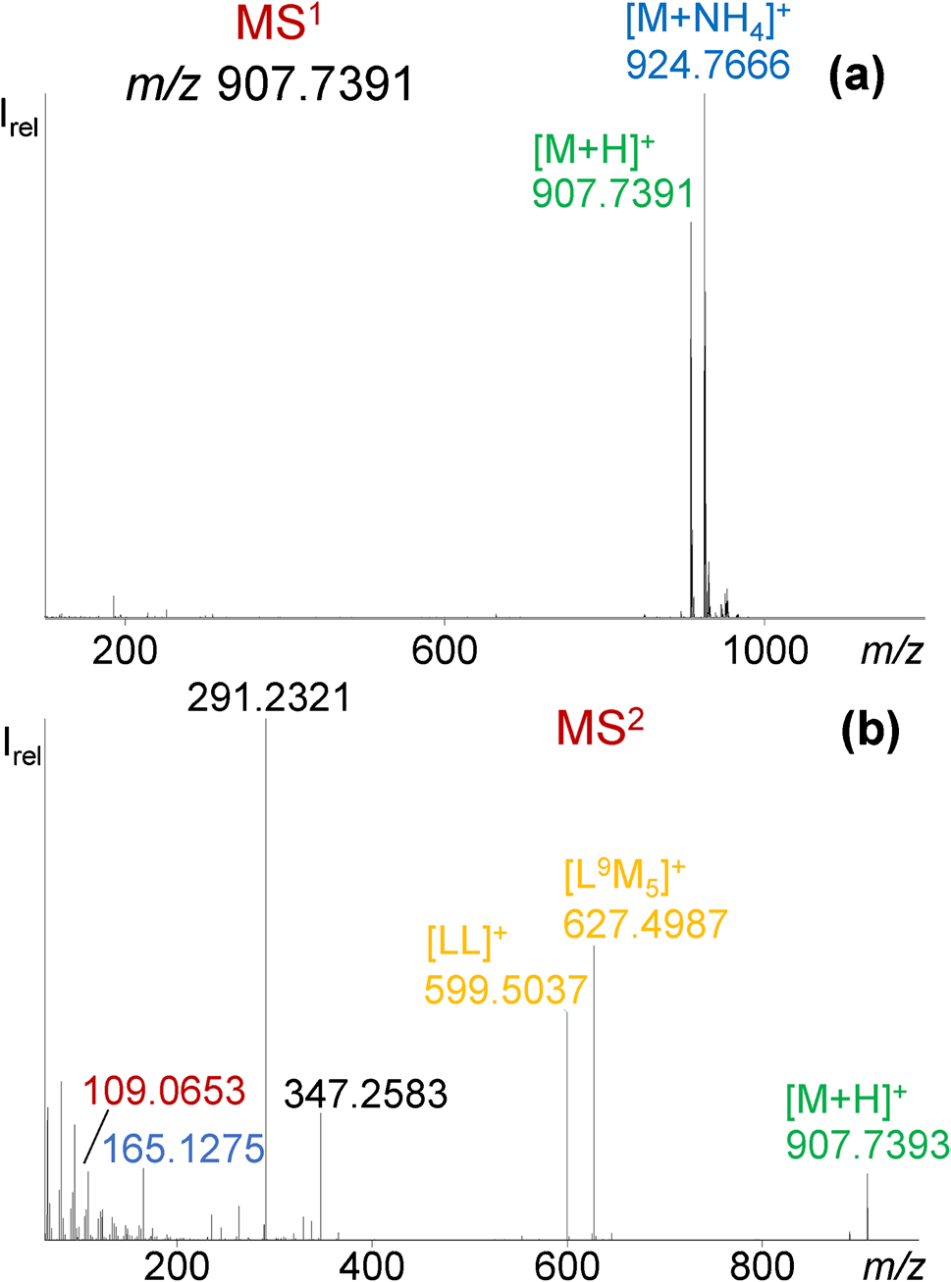
LC-Orbitrap-HRMS spectra of a synthesized LL^9^M_5_ standard mixture (four compounds) in (**a**) MS^1^ (first dimension) and (**b**) MS^2^ (second dimension) with characteristic fragment ions

FuFA-containing TAGs shared the HPLC elution range with TAGs featuring (only) conventional fatty acids. FuFAs with nine or eleven carbons in the α-connected carboxyalkyl chain, one or two methyl groups in β,β′-positions of the furan moiety, and three or five carbons in the alkyl chain in α′-position (Table 1) give rise to 2^3^ structural variants including two pairs of isomers, i.e., 11D3 and 9D5 as well as 11M3 and 9M5. These most prominent FuFAs feature 17 to 22 carbon atoms (Table 1). Nominal masses of FuFAs with 22 carbon atoms (e.g., 11D5, C_22_H_38_O_3_) are isobaric with convention 23:2 isomers (C_23_H_42_O_2_). First-dimension mass spectra (MS^1^) of TAGs only featured the quasi molecular ions [M + H]^+^ and—in varying abundance ratio—[M + NH_4_]^+^ ions, the latter due to the presence of ammonium acetate in the mobile phase (Fig. 2a) [27]. The molecular formulae of 11D5 and 23:2 isomers (O vs. CH_4_) differ by 0.0364 Da in their exact masses with the one of FuFAs being lower compared to those of conventional fatty acids (Table 3, Table S1).

**Table 3:** Mass differences between the same nominal masses for five examples

	Molecular formula	Exact mass (*m*/*z*)	Difference in formula	Exact mass (*m*/*z*)	Δ*m* (Da)
Nominal mass #1					
11D5	C_22_H_38_O_3_	350.2821	O	15.9949	− 0.0364
23:2	C_23_H_42_O_2_	350.3185	CH_4_	16.0313	0
Nominal mass #2					
[tri9M5 + H]^+^	C_60_H_99_O_9_	963.7289	O	15.9949	− 0.0364
[L^9^D_5_^9^D_5_ + H]^+^	C_61_H_103_O_8_	963.7653	CH_4_	16.0313	0
Nominal mass #3					
[OO^9^D_5_ + H]^+^	C_59_H_105_O_7_	925.7860	H_8_O	24.0575	− 0.0575
[OEpEp + H]^+^	C_61_H_97_O_6_	925.7285	C_2_	24.0000	0
Nominal mass #4					
[P^9^M_5_]^+^	C_38_H_67_O_5_	603.4989	O	15.9949	− 0.0364
[OO]^+^	C_39_H_71_O_4_	603.5353	CH_4_	16.0313	0
Nominal mass #5					
[O^9^D_5_]^+^	C_41_H_71_O_5_	643.5302	H_8_O	24.0575	− 0.0575
[EpEp]^+^	C_43_H_63_O_4_	643.4727	C_2_	24.0000	0

In agreement with that, other pairs with nominally isobaric [M + H]^+^ ions like tri9M5 ([C_60_H_99_O_9_ + H]^+^, *m/z* 963.7289) and L^9^D_5_^9^D_5_ ([C_61_H_102_O_8_ + H]^+^, *m/z* 963.7653) also differ by Δ*m* = 0.0364 Da (Table 3). However, fish oils are known to feature high shares of PUFAs with five or six double bonds such as docosahexaenoic acid (22:6Δ4,7,10,13,16,19, DHA; in TAGs: Dh) and eicosapentaenoic acid (20:5Δ5,8,11,14,17, EPA; in TAGs: Ep) (Table 2). The high number of double bonds in these ω-3 fatty acids leads to a second case where nominally isobaric ions differ by two carbon atoms (difference in the molecular formulae: C_2_ vs. H_8_O; Δ*m* = 0.0575 Da). For instance, Δ*m* of 0.0575 Da applies to [M + H]^+^ of the nominally isobaric pair OEpEp ([C_61_H_96_O_6_ + H]^+^, *m/z* 925.7285) and OO^9^D_5_ ([C_59_H_104_O_7_ + H]^+^, *m/z* 925.7860) (Table 3). The required mass resolutions of *R* = 26,500 (“O vs. CH_4_” case) and 16,100 (“C_2_ vs. H_8_O” case) are easily met with the present instrument.

Despite these distinct mass differences between nominally isobaric molecular ions, extraction of the exact masses of [M + H]^+^ or [M + NH_4_]^+^ of FuFA-containing TAGs in a fish oil from the LC-Orbitrap-HRMS chromatogram (mass accuracy, 4 ppm) did not facilitate an unequivocal detection in first-dimension mass spectra (MS^1^, Fig. S1). Clicking through the peak in MS^1^, the [M + H]^+^ ion (here: *m*/*z* 1019.7698) varied in abundance and was accompanied by about ten other, typically more abundant, peaks with other masses (Fig. S1b). Hence, the assignment of FuFA-containing TAGs by LC-Orbitrap-HRMS necessitated the involvement of MS^2^ data.

LC-MS^2^ spectra of TAGs (automatically generated by the instrument) are dominated by [M-RCOO]^+^ fragment ions (release of RCOOH from [M + H]^+^) (Fig. 2b) [27]. Purposefully synthesized TAGs with one, two, or three units of 9M5 and/or linoleic acid (18:2Δ9,12; L) [6] enabled us to confirm that FuFA-containing TAGs fragmented in the same way. Like [M + H]^+^ of TAGs, the nominally isobaric [OO]^+^ ([C_39_H_71_O_4_]^+^, *m/z* 603.5353) and [P^9^M_5_]^+^ ([C_38_H_67_O_5_]^+^, *m/z* 603.4989) [M-RCOO]^+^ fragment ions also differ by Δ*m* = 0.0364 Da (Table 3) while nominally isobaric pairs like [EpEp]^+^ ([C_43_H_63_O_4_]^+^, *m/z* 643.4727) and [O^9^D_5_]^+^ ([C_41_H_71_O_5_]^+^, *m/z* 643.5302) are separated by Δ*m* = 0.0575 Da (Table 3). The required mass resolutions of *R* = 16,680 and *R* = 11,190, respectively, are also met with the instrument.

However, screening for FuFA-containing [M-RCOO]^+^ fragment ions in MS^2^ spectra did not yet lead to an unequivocal assignment of a TAG, since a fragment ion such as [L^9^M_5_]^+^, for example, is formed from both LL^9^M_5_ and OL^9^M_5_. Therefore, filtering for FuFA-containing TAGs in MS^2^ should include [M + H]^+^ (e.g., *m*/*z* 907.7391 in case of LL^9^M_5_) (Fig. 2b).

The mass difference between theoretical and measured masses of [M + H]^+^ in MS^2^ was mostly < 2 ppm (Table 4). Loss of one fatty acid moiety from [M + H]^+^ of LL^9^M_5_ (standard) generated both *m*/*z* 599.5037 ([LL]^+^) and *m*/*z* 627.4987 ([L^9^M_5_]^+^). In addition, the corresponding MS^2^ spectrum featured the [RCO]^+^ fragment ion (formally [FuFA – OH]^+^) (here: *m*/*z* 291.2321 for 9M5) as the base peak along with the diagnostic allylic ion ([CH_2_(furan moiety)alkyl chain]^+^ of FuFAs: later furan core ion) (here: *m*/*z* 165.1275 for 9M5) (Fig. 1), and also the ion formed by McLafferty rearrangement of FuFAs (here: *m*/*z* 109.0653 for 9M5). The simultaneous occurrence of these three types of fragment ions in MS^2^ spectra verified the presence of a FuFA-containing TAG (here: 9M5 in LL^9^M_5_). The [RCO]^+^ fragment ion of FuFAs represented the base peak in the MS^2^ spectra of FuFA-containing TAGs but played only a subordinate role in TAGs with conventional fatty acids. Hence, it will be highlighted in the form of [FCO]^+^ in the following.

**Table 4:** Measured and calculated *m*/*z* values of exemplarily seven TAGs and the difference between these two values in [Da] and in [ppm]

	Measured [M + H]^+^	Calculated [M + H]^+^	Δ*m/z* [Da]	Δ*m/z* [ppm]
LLL	879.7458	879.7442	0.0016	1.819
OOO	885.7911	885.7912	0.0001	0.113
LL^9^M_5_	907.7380	907.7391	0.0011	1.212
OO^9^M_5_	911.7690	911.7704	0.0009	0.987
L^9^M_5_^9^M_5_	935.7332	935.7340	0.0008	0.855
O^9^M_5_^9^M_5_	937.7492	937.7497	0.0004	0.416
tri9M5	963.7283	963.7290	0.0013	1.307

Namely, FuFA-containing TAGs in LC-Orbitrap-HRMS spectra can be identified by measuring a low number of [FCO]^+^ fragment ions. Specifically, only six *m*/*z* values of [FCO]^+^ fragment ions were required for the analysis of eight relevant FuFAs (printed in bold), i.e., *m*/*z* 263.2011 (7M5, **9M3**), *m*/*z* 277.2168 (7D5, **9D3**), *m*/*z* 291.2324 (**9M5**, **11M3**) (Fig. 1), *m*/*z* 305.2481 (**9D5**, **11D3**), *m*/*z* 319.2637 (**11M5**, 13M3), and *m*/*z* 333.2794 (**11D5**, 13D3). Since each exact mass of the [FCO]^+^ fragment ion was formed by two FuFA isomers (Fig. 1), the list additionally covers four less relevant FuFAs with a shorter (7M5, 7D5) or longer (11M3, 13D3) carboxyalkyl chain. Additional, very rare alternatives of [FCO]^+^ fragment ions of FuFAs with 7 or 13 C-atoms in the carboxyalkyl chain are compiled in the supporting information (Table S2). Still, distinguishing these pairs of positional isomers required the implementation of additional fragment ions (see below).

### Method of LC-Orbitrap-HRMS detection of FuFA-containing TAGs in MS^2^

Initial filtering of FuFA-containing TAGs in MS^2^ spectra via [M + H]^+^ and [M + NH_4_]^+^ required the calculation of exact masses of potentially food-relevant FuFA-containing TAGs. In all but one case, [M + H]^+^ ions were more abundant than [M + NH_4_]^+^ ions; therefore, only the [M + H]^+^ ions were shown in the list (Table S3) of all combinations of six relevant FuFAs (9M5, 11M3, 9D5, 11D3, 11D5, 13D3) with seven characteristic conventional fatty acids in fish and plant oils, i.e., palmitic acid (16:0, P), oleic acid (18:1*n-9*, O), linoleic acid (18:2*n-6*, L), α-linolenic acid (18:3*n-3*, Ln), EPA (Ep), docosapentaenoic acid (22:5Δ7,10,13,16,19, DPA; in TAGs: Dp), and DHA (Dh). Due to the low abundance of FuFAs, it was reasonable to assume that, if present, (detectable) TAGs will mainly feature only one FuFA. Therefore, emphasis was put on TAGs consisting of one FuFA and two conventional fatty acids (Table S3). For the sake of completeness, examples of TAGs with two FuFAs are listed in the supporting information (Table S4).

### Filter for identification of FuFA-containing TAGs in LC-Orbitrap-HRMS spectra

**Fig. 3:**
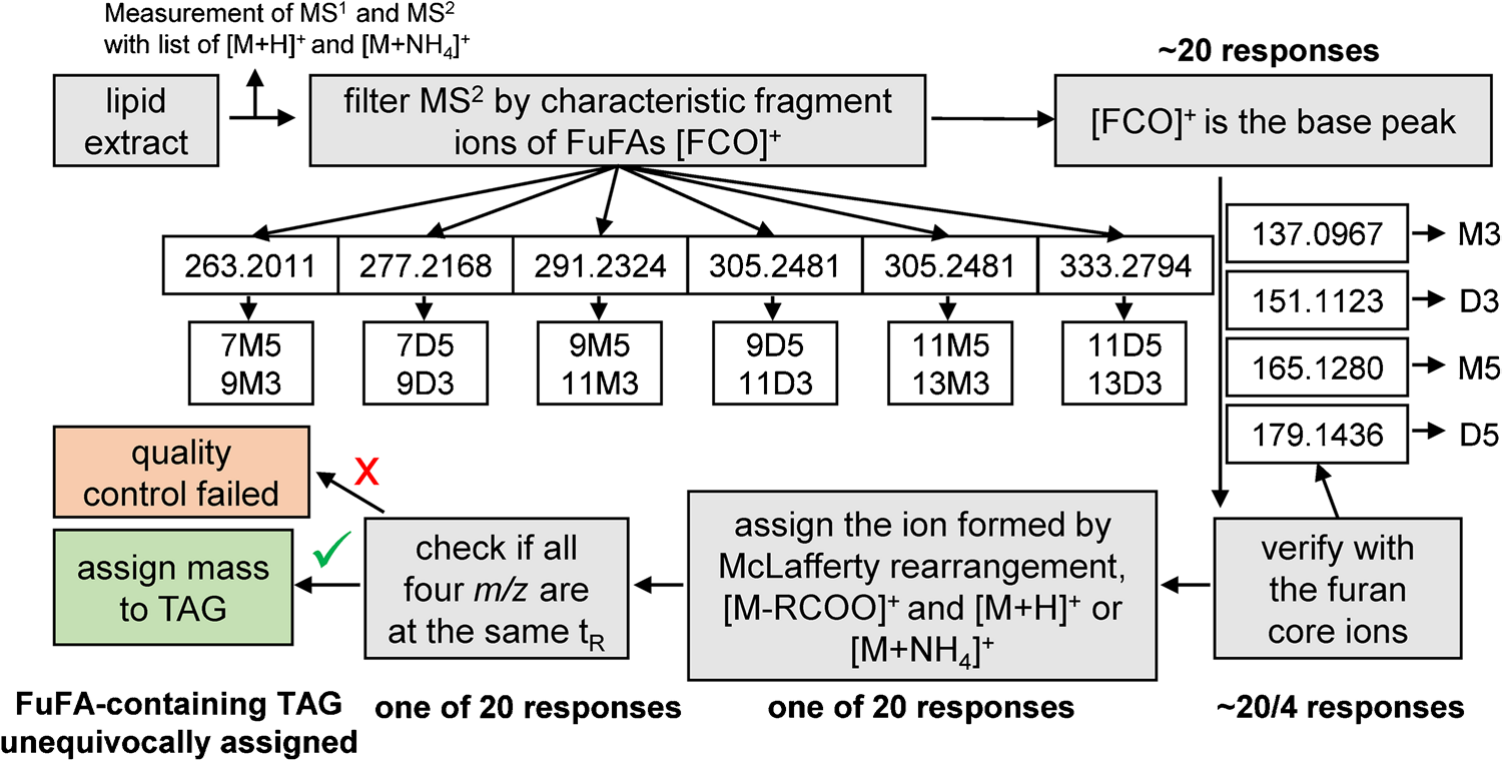
Flow chart of the identification process of FuFA-containing TAGs

**Fig. 4:**
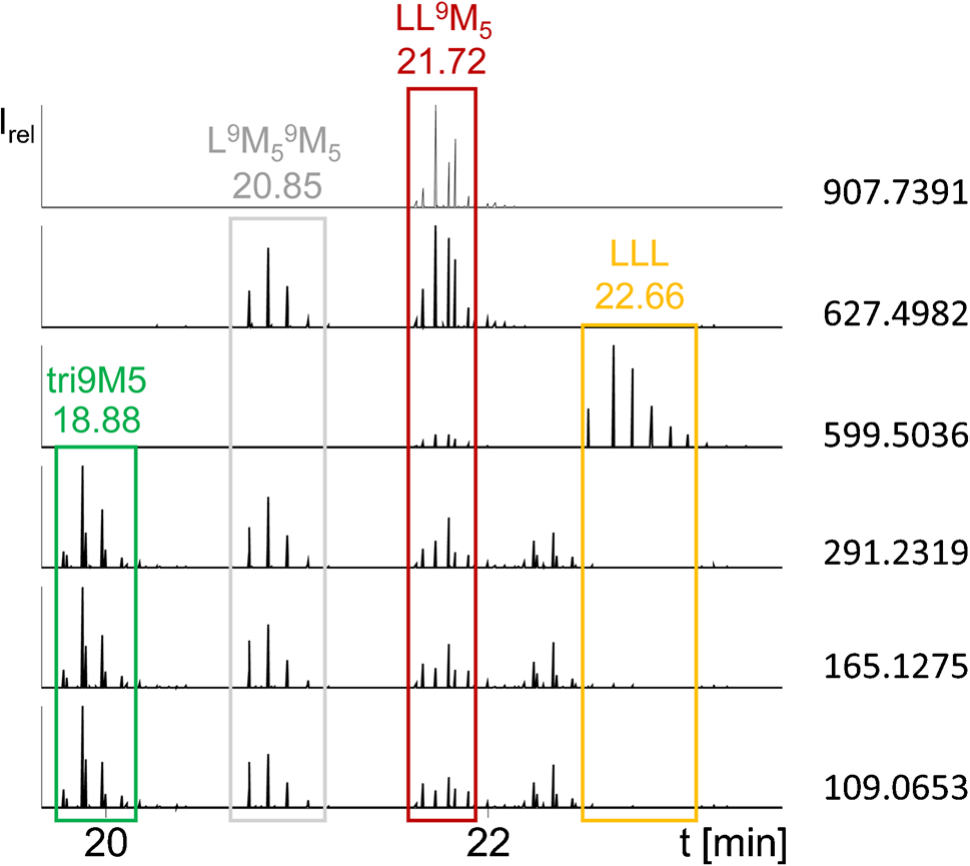
Filter of the MS^2^ mass spectra in LC-Orbitrap-HRMS measurements of four TAGs obtained from the synthesis of LLL and 9M5 [6]. Trigger points of the precursor ions are shown

In the following, [M + H]^+^ ions of FuFA-containing TAGs in MS^2^ (Table S3) were software-filtered (Thermo Xcalibur) in the presence of the [FCO]^+^ ion, the furan core ion, and the ion formed by McLafferty rearrangement (Figs. 3 and 4) allowing a maximum deviation of 4 ppm from the exact masses. Despite these precisely fitting selections, FuFA-containing TAGs could not be detected in case of an insufficient chromatographic separation of the sample. Apparently, overlaying highly abundant peaks from non-FuFA-TAGs inhibited the filter process. While this problem could be overcome by choosing a sufficiently good chromatographic separation (conditions are shown in the supporting information, Table S5), this problem is likely to exist in also other LC-HRMS studies where the selectivity is appropriate but filtering still fails.

Once this chromatographic problem was solved, the presence of a FuFA-containing TAG via [M + H]^+^ ion could be verified by means of the [FCO]^+^ ion in MS^2^ spectra and narrowed down to two positional isomers (e.g., #7 and #8, Table 5). In a second step, the concrete structure of the FuFA was derived from the *m*/*z* value of the furan core ions (e.g., #7, *m*/*z* 305.2481). Finally, the full structure of the FuFA-containing TAG could be determined by means of the combined presence of (i) [M-RCOO]^+^ fragment ions, (ii) ion formed by McLafferty rearrangement, and (iii) [M + H]^+^/[M + NH_4_]^+^ (Fig. 3). E.g., the presence of 9M5 in LL^9^M_5_ ([M + H]^+^, *m*/*z* 907.7391, Table 5, #3) was verified by the base peak at *m*/*z* 291.2324 ([FCO]^+^) along with *m*/*z* 165.1280 (furan core ion) and *m*/*z* 109.0654 (ion formed by McLafferty rearrangement), while the [M-RCOO]^+^ fragment ions *m*/*z* 627.4989 and 599.5039 ([L^9^M_5_]^+^ and [LL]^+^, respectively) verified the presence of two L moieties in the FuFA-containing TAG (Fig. 2b).

**Table 5:** List of TAGs and FuFA-containing TAGs with the molecular formula for [M + H]^+^ that are mentioned in the paper

TAG	No	Molecular formula	[M + H]^+^	FuFA-specific base peak ([FCO]^+^)* in MS^2^
PL^9^D_3_	1	C_55_H_97_O_7_	869.7235	277.2168
OL^9^D_3_	2	C_57_H_99_O_7_	895.7391	277.2168
LL^9^M_5_	3	C_58_H_99_O_7_	907.7391	291.2324
OL^9^M_5_	4	C_58_H_101_O_7_	909.7548	291.2324
OO^9^M_5_	5	C_58_H_103_O_7_	911.7704	291.2324
LL^9^D_5_	6	C_59_H_101_O_7_	921.7548	305.2481
OL^9^D_5_	7	C_59_H_103_O_7_	923.7704	305.2481
PLn^11^D_5_	8	C_59_H_103_O_7_	923.7704	333.2794
OEpEp	9	C_61_H_96_O_6_	925.7285	–
OO^9^D_5_	10	C_59_H_105_O_7_	925.7861	305.2481
OL^11^D_5_	11	C_61_H_107_O_7_	951.8017	333.2794
tri9M5	12	C_60_H_99_O_9_	963.7290	291.2324
L^9^D_5_^9^D_5_	13	C_61_H_103_O_8_	963.7653	305.2481
EpEp^9^M_5_	14	C_62_H_95_O_7_	951.7078	291.2324
EpEp^11^D_3_	15	C_63_H_97_O_7_	965.7235	305.2481
EpDh^11^D_5_	16	C_67_H_103_O_7_	1019.7704	333.2794

^*^[FCO]^+^ formally denotes the [FuFA-OH]^+^ ion formed by α-cleavage

Since only primary fatty acids are removed from TAGs, either one [M-RCOO]^+^ ion (in the case of the same fatty acids in *sn*-1 and *sn*-3 position) or two [M-RCOO]^+^ ions can result from this fragmentation. Specifically, LL^9^M_5_ will generate [LL]^+^ and [L^9^M_5_]^+^ while L^9^M_5_L will (almost) exclusively generate [L^9^M_5_]^+^. Since LL^9^M_5_ and L^9^M_5_L will likely co-elute in HPLC, the presence of only LL^9^M_5_ or both positional isomers is difficult to determine if [LL]^+^ and [L^9^M_5_]^+^ are detected in MS^2^. For the reason of simplicity, TAGs were named according to a specific scheme, mentioned at the beginning.

### Application of the method to FuFA-containing TAGs in king oyster mushroom (*Pleurotus eryngii*)

LC-Orbitrap**-**MS^2^-based filtering of a king oyster mushroom (*Pleurotus eryngii*) sample according to Table S3 and the follow-up steps described above enabled the identification of 18 FuFA-containing TAGs (Table 6). In agreement with a predominance of 9D5 >> 9M5 > 7D5, these FuFAs were present in 13, 3, and 2 TAGs, respectively. Similarly, the conventional fatty acids were represented by the most relevant of mushrooms, namely linoleic acid (18:2*n-6*, L) >> oleic acid (18:1*n-9*, O) > palmitic acid (16:0, P) [28]. Specifically, the most prominent peak originated from LL^9^D_5_ followed by LO^9^D_5_. In the MS^2^ spectrum, presence of LL^9^D_5_ ([M + H]^+^ at *m*/*z* 921.7543) was verified by the base peak (*m*/*z* 305.2479), furan core ion (*m*/*z* 179.1430), and the [M-RCOO]^+^ fragment ions at *m*/*z* 599.5035 ([LL]^+^) and 641.5142 ([L^9^D_5_]^+^) (Table 6). Presence of other FuFA-containing TAGs in the sample was verified the same way.

**Table 6:** FuFA-containing TAGs identified in king oyster mushroom (*Pleurotus eryngii*) extract

TAG*	*t*_R_ [min]	Molecular formula	[M + H]^+^	Fragment ions**	Carbon number	Signal intensity (*I*)***
XY^9^D_5_****	20.80	C_57_H_96_O_7_	893.7233	123.0808 179.1432 **305.2478**	54	570
LL^7^D_5_	20.80	C_57_H_96_O_7_	893.7233	123.0808 599.5031 179.1432 613.4835 **277.2165**	54	284
15:0-Ln^9^D_5_	21.65	C_56_H_96_O_7_	881.7230	123.0808 559.4725 179.1432 601.4830 **305.2479 **639.4975	53	1010
LL^9^M_5_	21.66	C_58_H_98_O_7_	907.7379	109.0653 599.5036 165.1276 627.4988 **291.2321**	55	230
L^9^D_5_^9^D_5_	21.89	C_61_H_102_O_8_	963.7649	123.0808 641.5142 179.1431 683.5248 **305.2480**	58	9980
PnL^9^D_5_	22.03	C_57_H_98_O_7_	895.7386	123.0807 573.4880 179.1431 615.4988 **305.2478 **641.5142	54	2110
LO^7^D_5_	22.14	C_57_H_98_O_7_	895.7384	123.0807 601.5197 179.1431 613.4827 **277.2164 **615.4984	54	278
LL^9^D_5_	22.24	C_59_H_100_O_7_	921.7543	123.0807 599.5035 179.1431 641.5142 **305.2479**	56	66,800
15:0-L^9^D_5_	22.83	C_56_H_98_O_7_	883.7387	123.0808 561.4880 179.1431 603.4987 **305.2479 **641.5143	53	3770
LO^9^M_5_	23.30	C_58_H_100_O_7_	909.7537	109.0653 601.5195 165.1275 627.4985 **291.2321 **629.5150	55	339
O^9^D_5_^9^D_5_	23.34	C_61_H1_04_O_8_	965.7803	123.0808 643.5302 179.1431 683.5249 **305.2478**	58	1430
LO^9^D_5_	23.71	C_59_H_102_O_7_	923.7696	123.0807 601.5192 179.1430 641.5139 **305.2478 **643.5297	56	29,100
15:0-O^9^D_5_	24.34	C_56_H_100_O_7_	885.7546	123.0808 563.5038 179.1432 603.4987 **305.2478 **643.5302	53	1280
17:0-L^9^D_5_	24.59	C_58_H_102_O_7_	911.7698	123.0807 589.5192 179.1432 631.5297 **305.2477 **641.5143	55	815
OO^9^M_5_	24.69	C_58_H_102_O_7_	911.7711	109.0653 603.5348 165.1274 629.5142 **291.2321**	55	121
PO^9^D_5_	25.13	C_57_H_102_O_7_	899.7696	123.0807 577.5192 179.1431 617.5142 **305.2479 **643.5298	54	9550
OO^9^D_5_	25.18	C_59_H_104_O_7_	925.7855	123.0807 603.5349 179.1431 643.5300 **305.2479**	56	10,100
OS^9^D_5_	26.80	C_59_H_106_O_7_	927.8011	123.0808 605.5505 179.1431 643.5300 **305.2477 **645.5455	56	977

^*^Fatty acids are listed as follows: first two conventional fatty acids with the one with higher mass listed first, followed by the FuFA in position three of the glycerol backbone. This order must not reflect the correct order of fatty acids

^**^From top to bottom: on the left: ion formed by McLafferty rearrangement, furan core ion, base peak ([FCO]+)—formally [FuFA-OH]+, printed in bold), and on the right: finally two or three [R-COOH]+ ions

^***^Signal intensity of the base peak, divided by a factor of 1000. In the case of FuFA-containing TAGs, this is the [FCO]^+^ ion (formally [FuFA-OH]^+^, formed by α-cleavage)

^****^XY stands for two conventional fatty acids that could not be clearly assigned. Based on the characteristic fragment ions, the FuFA could be assigned

The abundance ratio between the most abundant (LL^9^D_5_, intensity (*I*) = 66,800) and the least abundant (OO^9^M_5_, *I* = 121) FuFA-containing TAG was spread over more than two orders of magnitude (Table 6). Still, OO^9^M_5_ could be assigned with the LC-Orbitrap-HRMS method. However, structural assignments were more difficult for FuFA-containing TAGs due to their low abundance in the sample. Co-elutions (see the first two TAGs in Table 6) still enabled the assignment of the characteristic [FCO]^+^ fragment ion but not of the corresponding [M-RCOO]^+^ fragment ions. Hence, only the FuFA but not the conventional fatty acids could be identified in the TAG (see the first example, Table 6).

### FuFA-containing TAGs in two fish oil samples

The fish oil I was dominated by the conventional fatty acids EPA and DHA and FuFAs 11D3, 11D5, and 9M5 [18]. Combinations of these fatty acids were found in nine of the thirteen FuFA-containing TAGs that could be detected in the sample (Table S6). Four TAGs contained 9M5 with the most abundant one (EpEp^9^M_5_) having an intensity of *I* = 2820 (Table S6). Representatives containing 11D3 (*n* = 4) were less abundant (maximum *I* = 626 for EpEp^11^D_3_) while the remaining five TAGs, topped by EpDh^11^D_5_ with *I* = 12,000, featured 11D5 (Table S6).

The fatty acid pattern of fish oil II (gilthead liver oil) was dominated by 16:0, 18:1*n-9*, 18:2*n-6*, and stearic acid (18:0) while EPA and DHA were virtually absent (Table S7). Interestingly, 11D5 was only present at traces in one FuFA-containing TAG (OL^11^D_5_, *I* = 29), while three TAGs, respectively, featured 9M5, 9D3, and 7D5. The most prominent peaks originated from OL^9^D_3_ (*I* = 3,920) and PL^9^D_3_ (*I* = 2,690) (Table S7).

## Method information

The reproducibility of the method was tested by three injections of fish oil II (gilthead liver oil) and comparison of the relative abundances of four TAGs and five FuFA-containing TAGs (Table S8). The most abundant non-FuFA and FuFA-containing TAG of the first run was used as the reference (100%). Based on these data, conventional (and more abundant) TAGs showed good reproducibility (10–15% relative STDEV) with the exception of OOO due to its low relative abundance in run 3 (Table S8). Compared to that, relative standard deviations of FuFA-containing TAGs were in the range of 12–37% which was deemed acceptable given the fact that abundances were lower.

The limit of detection (LOD) of FuFA-containing TAGs could not be directly determined due to the lack both of reference standards and MSMS responses. Hence, it was aimed to estimate the minimum amount of a FuFA-containing TAG indirectly from previous data in the corresponding samples which were obtained after transmethylation and determination of the FuFAs as fatty acid methyl esters (FAMEs). Specifically, the king oyster mushroom was found to contain 33 mg 9D5/100 g fungi dry weight (determined as FAME) [29]. Based on the prerequisites that (i) all 9D5 in the mushroom was stored in the TAG fraction and that (ii) LC-Orbitrap-MSMS responses of all FuFA-containing TAGs were similar (which was not unlikely due to the formation of the highly abundant ion formed via McLafferty rearrangement), we could calculate the individual contribution of FuFA-containing TAGs from the peak areas and related them to the reported sum value (Table S9). According to that, the minimum amount that could be determined in a FuFA-containing TAG of the mushroom was estimated at 0.1 mg/100 g dry weight (Table S9). Based on a lipid content of ~ 5% lipids (estimated) in the dry weight of the mushroom, this corresponds with the lowest detectable amount of 2 mg/100 g lipids FuFA-containing TAG.

In the same way, we also estimated the content of 9M5, 11D3, and 11D5 in fish oil I with a FuFA amount of 1030 mg/100 g lipids as determined via methyl esters [18]. Accordingly, the lowest measurable contribution originated from EpDp^11^D_5_ at 4.6 mg/100 g lipids (Table S10).

Overall, the minimum amount that could be estimated in this way in two samples was in the same range of 1–10 mg FuFA-containing TAGs/100 g lipids. This concentration range may be considered in the planning of further studies on FuFA-containing TAGs.

## Conclusion

The present LC-Orbitrap-HRMS screening method allowed the identification of 39 different FuFA-containing TAGs in three samples. The method can be easily adopted and applied to other sample matrices. Such applications will support the overall evaluation of the value of FuFAs and their role as natural antioxidants which is currently underexplored.

## Supplementary Information

Below is the link to the electronic supplementary material.

Supplementary file1 (PDF 341 KB)

## Data Availability

The datasets generated and/or analyzed during the current study are available from the corresponding author on reasonable request.
