# Supplementary material for: LC-Orbitrap-HRMS method for analysis of traces of triacylglycerols featuring furan fatty acids
Source: Anal Bioanal Chem. 2022 Dec 20;415(5):875–85. doi: 10.1007/s00216-022-04480-y (PMC9883336; doi:10.1007/s00216-022-04480-y)
Supplement: Supplementary file 1 — Supplementary file1 (PDF 341 KB) [file 216_2022_4480_MOESM1_ESM.pdf]

Supporting Information:

LC-Orbitrap-HRMS method for analysis of traces of triacylglycerols featuring furan fatty acids

Nina Wiedmaier-Czerny[1], Walter Vetter[1]*

[1] Institute of Food Chemistry, Department of Food Chemistry (170b), University of Hohenheim, D-70593 Stuttgart, Germany

* Corresponding authors:

Walter Vetter

Phone: +49 711 459 24016

Fax: +49 711 459 24377

E-Mail: walter.vetter@uni-hohenheim.de

Tables

Table S1: Mass differences between the same nominal masses for two examples

	molecular formula	exact mass	difference in formula	exact mass	Δm
$[tri9M5+NH_4]^+$	$C_{60}H_{102}O_9N$	980.7555	O	15.9949	-0.0364
$[L^9D_5{}^9D_5+NH_4]^+$	$C_{61}H_{106}O_8N$	980.7919	CH_4	16.0313	0
$[OO^9D_5+NH_4]^+$	$C_{59}H_{108}O_7N$	942.8126	H_8O	24.0575	-0.0575
$[OEpEp+NH_4]^+$	$C_{61}H_{100}O_6N$	942.7551	C_2	24.0000	0

Table S2: $[FCO]^+$ ions (formally denotes the $[FuFA\text{-}OH]^+$ ion formed by α-cleavage) of FuFAs that are not mentioned in the main text.

**FuFA**	**$[FCO]^+$**
7M3	235.1698
7D3	249.1855
13M5	347.2950
13D5	361.3107

Table S3: List of TAGs and FuFA-containing TAGs with the molecular formula for $[M+H]^+$ and the FuFA-specific base peak ($[FCO]^+$) in MS^2. The used FuFAs are the most common FuFAs.

TAG *	No.	molecular formula	$[M+H]^+$	FuFA-specific base peak ($[FCO]^+$) in MS^2
PPP	1	$C_{51}H_{99}O_6$	807.7442	
PnPn^{9}M$_5$	2	$C_{54}H_{95}O_7$	855.7078	291.2324
PnP^{9}M$_5$	3	$C_{54}H_{97}O_7$	857.7235	291.2324
PP^{9}M$_5$	4	$C_{54}H_{99}O_7$	859.7391	291.2324
PnPn^{9}D$_5$	5	$C_{55}H_{97}O_7$	869.7235	305.2481
PnP^{9}D$_5$	6	$C_{55}H_{99}O_7$	871.7391	305.2481
LnLnLn	7	$C_{57}H_{93}O_6$	873.6973	
PP^{9}D$_5$	8	$C_{55}H_{101}O_7$	873.7548	305.2481
LLL	9	$C_{57}H_{99}O_6$	879.7442	
PnLn^{9}M$_5$	10	$C_{56}H_{95}O_7$	879.7078	291.2324
PLn^{9}M$_5$	11	$C_{56}H_{97}O_7$	881.7235	291.2324
PnL^{9}M$_5$	12	$C_{56}H_{97}O_7$	881.7235	291.2324
PL^{9}M$_5$	13	$C_{56}H_{99}O_7$	883.7391	291.2324
PnO^{9}M$_5$	14	$C_{56}H_{99}O_7$	883.7391	291.2324
PnS^{9}M$_5$	15	$C_{56}H_{101}O_7$	885.7548	291.2324
PO^{9}M$_5$	16	$C_{56}H_{101}O_7$	885.7548	291.2324
OOO	17	$C_{57}H_{105}O_6$	885.7912	
PS^{9}M$_5$	18	$C_{56}H_{103}O_7$	887.7704	291.2324
PnLn^{9}D$_5$	19	$C_{57}H_{97}O_7$	893.7235	305.2481
PLn^{9}D$_5$	20	$C_{57}H_{99}O_7$	895.7391	305.2481
PnL^{9}D$_5$	21	$C_{57}H_{99}O_7$	895.7391	305.2481
LnEp^{9}D$_5$	22	$C_{57}H_{99}O_7$	895.7391	305.2481
PL^{9}D$_5$	23	$C_{57}H_{101}O_7$	897.7548	305.2481
PnO^{9}D$_5$	24	$C_{57}H_{101}O_7$	897.7548	305.2481
LEp^{9}D$_5$	25	$C_{57}H_{101}O_7$	897.7548	305.2481
PnPn^{11}D$_5$	26	$C_{57}H_{101}O_7$	897.7548	333.2794
PnP^{11}D$_5$	27	$C_{57}H_{103}O_7$	899.7704	333.2794
PnS^{9}D$_5$	28	$C_{57}H_{103}O_7$	899.7704	305.2481
PO^{9}D$_5$	29	$C_{57}H_{103}O_7$	899.7704	305.2481
OEp^{9}D$_5$	30	$C_{57}H_{103}O_7$	899.7704	305.2481
SEp^{9}D$_5$	31	$C_{57}H_{105}O_7$	901.7861	305.2481
PS^{9}D$_5$	32	$C_{57}H_{105}O_7$	901.7861	305.2481
PP^{11}D$_5$	33	$C_{57}H_{105}O_7$	901.7861	333.2794
PnEp^{9}M$_5$	34	$C_{58}H_{95}O_7$	903.7078	291.2324
LnLn^{9}M$_5$	35	$C_{58}H_{95}O_7$	903.7078	291.2324
LnL^{9}M$_5$	36	$C_{58}H_{97}O_7$	905.7235	291.2324
PEp^{9}M$_5$	37	$C_{58}H_{97}O_7$	905.7235	291.2324
LL^{9}M$_5$	38	$C_{58}H_{99}O_7$	907.7391	291.2324
LnO^{9}M$_5$	39	$C_{58}H_{99}O_7$	907.7391	291.2324
LnS^{9}M$_5$	40	$C_{58}H_{101}O_7$	909.7548	291.2324
LO^{9}M$_5$	41	$C_{58}H_{101}O_7$	909.7548	291.2324
LS^{9}M$_5$	42	$C_{58}H_{103}O_7$	911.7704	291.2324
OO^{9}M$_5$	43	$C_{58}H_{103}O_7$	911.7704	291.2324
OS^{9}M$_5$	44	$C_{58}H_{105}O_7$	913.7861	291.2324
SS^{9}M$_5$	45	$C_{58}H_{107}O_7$	915.8017	291.2324
LnS^{9}D$_5$	46	$C_{59}H_{95}O_7$	915.7078	305.2481
LS^{9}D$_5$	47	$C_{59}H_{97}O_7$	917.7235	305.2481
PnEp^{9}D$_5$	48	$C_{59}H_{97}O_7$	917.7235	305.2481
LnLn^{9}D$_5$	49	$C_{59}H_{97}O_7$	917.7235	305.2481
PEp^{9}D$_5$	50	$C_{59}H_{99}O_7$	919.7391	305.2481
OS^{9}D$_5$	51	$C_{59}H_{99}O_7$	919.7391	305.2481

LnL^{9}D$_{5}$	52	$C_{59}H_{99}O_{7}$	919.7391	305.2481
PnLn^{11}D$_{5}$	53	$C_{59}H_{101}O_{7}$	921.7548	333.2794
SS^{9}D$_{5}$	54	$C_{59}H_{101}O_{7}$	921.7548	305.2481
LnO^{9}D$_{5}$	55	$C_{59}H_{101}O_{7}$	921.7548	305.2481
LL^{9}D$_{5}$	56	$C_{59}H_{101}O_{7}$	921.7548	305.2481
LO^{9}D$_{5}$	57	$C_{59}H_{103}O_{7}$	923.7704	305.2481
PLn^{11}D$_{5}$	58	$C_{59}H_{103}O_{7}$	923.7704	333.2794
PnL^{11}D$_{5}$	59	$C_{59}H_{103}O_{7}$	923.7704	333.2794
OO^{9}D$_{5}$	60	$C_{59}H_{105}O_{7}$	925.7861	305.2481
PL^{11}D$_{5}$	61	$C_{59}H_{105}O_{7}$	925.7861	333.2794
PnO^{11}D$_{5}$	62	$C_{59}H_{105}O_{7}$	925.7861	333.2794
PO^{11}D$_{5}$	63	$C_{59}H_{107}O_{7}$	927.8017	333.2794
LnEp^{9}M$_{5}$	64	$C_{60}H_{95}O_{7}$	927.7078	291.2324
PnS^{11}D$_{5}$	65	$C_{59}H_{107}O_{7}$	927.8017	333.2794
PS^{11}D$_{5}$	66	$C_{59}H_{109}O_{7}$	929.8174	333.2794
LEp^{9}M$_{5}$	67	$C_{60}H_{97}O_{7}$	929.7235	291.2324
OEp^{9}M$_{5}$	68	$C_{60}H_{99}O_{7}$	931.7391	291.2324
PDh^{9}M$_{5}$	69	$C_{60}H_{99}O_{7}$	931.7391	291.2324
SEp^{9}M$_{5}$	70	$C_{60}H_{101}O_{7}$	933.7548	291.2324
PnDh^{9}M$_{5}$	71	$C_{60}H_{101}O_{7}$	933.7548	291.2324
PDp^{9}M$_{5}$	72	$C_{60}H_{101}O_{7}$	933.7548	291.2324
PnDp^{9}M$_{5}$	73	$C_{60}H_{103}O_{7}$	935.7704	291.2324
PDh^{9}D$_{5}$	74	$C_{61}H_{101}O_{7}$	941.7235	305.2481
PnDh^{9}D$_{5}$	75	$C_{61}H_{99}O_{7}$	943.7391	305.2481
PnDp^{9}D$_{5}$	76	$C_{61}H_{101}O_{7}$	945.7548	305.2481
LnLn^{11}D$_{5}$	77	$C_{61}H_{101}O_{7}$	945.7548	333.2794
PnEp^{11}D$_{5}$	78	$C_{61}H_{101}O_{7}$	945.7548	333.2794
LnL^{11}D$_{5}$	79	$C_{61}H_{103}O_{7}$	947.7704	333.2794
PDp^{9}D$_{5}$	80	$C_{61}H_{103}O_{7}$	947.7704	305.2481
PEp^{11}D$_{5}$	81	$C_{61}H_{103}O_{7}$	947.7704	333.2794
LnO^{11}D$_{5}$	82	$C_{61}H_{105}O_{7}$	949.7861	333.2794
LL^{11}D$_{5}$	83	$C_{61}H_{105}O_{7}$	949.7861	333.2794
EpEp^{9}M$_{5}$	84	$C_{62}H_{95}O_{7}$	951.7078	291.2324
LnS^{11}D$_{5}$	85	$C_{61}H_{107}O_{7}$	951.8017	333.2794
LO^{11}D$_{5}$	86	$C_{61}H_{107}O_{7}$	951.8017	333.2794
OO^{11}D$_{5}$	87	$C_{61}H_{109}O_{7}$	953.8174	333.2794
LnDh^{9}M$_{5}$	88	$C_{62}H_{97}O_{7}$	953.7235	291.2324
LS^{11}D$_{5}$	89	$C_{61}H_{109}O_{7}$	953.8174	333.2794
OS^{11}D$_{5}$	90	$C_{61}H_{111}O_{7}$	955.8330	333.2794
LDh^{9}M$_{5}$	91	$C_{62}H_{99}O_{7}$	955.7391	291.2324
LnDp^{9}M$_{5}$	92	$C_{62}H_{99}O_{7}$	955.7391	291.2324
SS^{11}D$_{5}$	93	$C_{61}H_{113}O_{7}$	957.8487	333.2794
ODh^{9}M$_{5}$	94	$C_{62}H_{101}O_{7}$	957.7548	291.2324
LDp^{9}M$_{5}$	95	$C_{62}H_{101}O_{7}$	957.7548	291.2324
ODp^{9}M$_{5}$	96	$C_{62}H_{103}O_{7}$	959.7704	291.2324
SDh^{9}M$_{5}$	97	$C_{62}H_{103}O_{7}$	959.7704	291.2324
SDp^{9}M$_{5}$	98	$C_{62}H_{105}O_{7}$	961.7861	291.2324
tri9M5	99	$C_{60}H_{99}O_{9}$	963.7290	291.2324
EpEp^{9}D$_{5}$	100	$C_{63}H_{97}O_{7}$	965.7235	305.2481
LnDh^{9}D$_{5}$	101	$C_{63}H_{99}O_{7}$	967.7391	305.2481
LDh^{9}D$_{5}$	102	$C_{63}H_{101}O_{7}$	969.7548	305.2481
LnEp^{11}D$_{5}$	103	$C_{63}H_{101}O_{7}$	969.7548	333.2794
LnDp^{9}D$_{5}$	104	$C_{63}H_{101}O_{7}$	969.7548	305.2481
LEp^{11}D$_{5}$	105	$C_{63}H_{103}O_{7}$	971.7704	333.2794
ODh^{9}D$_{5}$	106	$C_{63}H_{103}O_{7}$	971.7704	305.2481
LDp^{9}D$_{5}$	107	$C_{63}H_{103}O_{7}$	971.7704	305.2481
PnDh^{11}D$_{5}$	108	$C_{63}H_{103}O_{7}$	971.7704	333.2794
OEp^{11}D$_{5}$	109	$C_{63}H_{105}O_{7}$	973.7861	333.2794
PDh^{11}D$_{5}$	110	$C_{63}H_{105}O_{7}$	973.7861	333.2794

SDh^9D_5	111	$C_{63}H_{105}O_7$	973.7861	305.2481
ODp^9D_5	112	$C_{63}H_{105}O_7$	973.7861	305.2481
$PnDp^{11}D_5$	113	$C_{63}H_{105}O_7$	973.7861	333.2794
$PDp^{11}D_5$	114	$C_{63}H_{107}O_7$	975.8017	333.2794
SDp^9D_5	115	$C_{63}H_{107}O_7$	975.8017	305.2481
$SEp^{11}D_5$	116	$C_{63}H_{107}O_7$	975.8017	333.2794
$EpDh^9M_5$	117	$C_{64}H_{97}O_7$	977.7235	291.2324
$EpDp^9M_5$	118	$C_{64}H_{99}O_7$	979.7391	291.2324
$EpDh^9D_5$	119	$C_{65}H_{99}O_7$	991.7391	305.2481
$EpEp^{11}D_5$	120	$C_{65}H_{101}O_7$	993.7548	333.2794
$EpDp^9D_5$	121	$C_{65}H_{101}O_7$	993.7548	305.2481
$EpEp^{11}D_5$	122	$C_{65}H_{101}O_7$	993.7548	333.2794
$LnDh^{11}D_5$	123	$C_{65}H_{103}O_7$	995.7704	333.2794
$LDh^{11}D_5$	124	$C_{65}H_{105}O_7$	997.7861	333.2794
$LnDp^{11}D_5$	125	$C_{65}H_{105}O_7$	997.7861	333.2794
$ODh^{11}D_5$	126	$C_{65}H_{107}O_7$	999.8017	333.2794
$LDp^{11}D_5$	127	$C_{65}H_{107}O_7$	999.8017	333.2794
$ODp^{11}D_5$	128	$C_{65}H_{109}O_7$	1001.8174	333.2794
$SDp^{11}D_5$	129	$C_{65}H_{111}O_7$	1003.8330	333.2794
$SDh^{11}D_5$	130	$C_{65}H_{109}O_7$	1001.8174	333.2794
$DhDh^9M_5$	131	$C_{66}H_{99}O_7$	1003.7391	291.2324
$DhDp^9M_5$	132	$C_{66}H_{101}O_7$	1005.7548	291.2324
tri9D5	133	$C_{63}H_{105}O_9$	1005.7759	305.2481
$DpDp^9M_5$	134	$C_{66}H_{103}O_7$	1007.7704	291.2324
$DhDh^9D_5$	135	$C_{67}H_{101}O_7$	1017.7548	305.2481
$DhDp^9D_5$	136	$C_{67}H_{103}O_7$	1019.7704	305.2481
$DhEp^{11}D_5$	137	$C_{67}H_{103}O_7$	1019.7704	333.2794
$EpDp^{11}D_5$	138	$C_{67}H_{105}O_7$	1021.7861	333.2794
$DpDp^9D_5$	139	$C_{67}H_{105}O_7$	1021.7861	305.2481
$DhDh^{11}D_5$	140	$C_{69}H_{105}O_7$	1045.7861	333.2794
$DhDp^{11}D_5$	141	$C_{69}H_{107}O_7$	1047.8017	333.2794
$DpDp^{11}D_5$	142	$C_{69}H_{109}O_7$	1049.8174	333.2794
tri11D5	143	$C_{69}H_{117}O_9$	1089.8698	333.2794

* fatty acids are listed as follows: first two, conventional fatty acids listed with increasing molecular mass followed by the FuFA in position three of the glycerol backbone. This order must not reflect the correct order of fatty acids.

Table S4: List of TAGs containing two FuFAs with the molecular formula for $[M+H]^+$ and the FuFA-specific base peak ($[FCO]^+$) in MS^2. The used three FuFAs are the most common FuFAs.

TAG *	No.	molecular formula	$[M+H]^+$	FuFA-specific base peak ($[FCO]^+$) in MS^2
$Pn^9M_5{}^9M_5$	1	$C_{57}H_{97}O_8$	909.7184	291.2324
$P^9M_5{}^9M_5$	2	$C_{57}H_{99}O_8$	911.7340	291.2324
$Pn^9M_5{}^9D_5$	3	C58H99O8	923.7340	291.2324/ 305.2481
$P^9M_5{}^9D_5$	4	C58H101O8	925.7497	291.2324/ 305.2481
$Ln^9M_5{}^9M_5$	5	$C_{59}H_{97}O_8$	933.7184	291.2324
$L^9M_5{}^9M_5$	6	$C_{59}H_{99}O_8$	935.7340	291.2324
$O^9M_5{}^9M_5$	7	$C_{59}H_{101}O_8$	937.7497	291.2324
$Pn^9D_5{}^9D_5$	8	C59H101O8	937.7497	305.2481
$S^9M_5{}^9M_5$	9	$C_{59}H_{103}O_8$	939.7653	291.2324
$P^9D_5{}^9D_5$	10	C59H103O8	939.7653	305.2481
$Ln^9M_5{}^9D_5$	11	C60H99O8	947.7340	291.2324/ 305.2481
$L^9M_5{}^9D_5$	12	C60H101O8	949.7497	291.2324/ 305.2481
$Pn^9M_5{}^{11}D_5$	13	C60H103O8	951.7653	291.2324/ 333.2794
$O^9M_5{}^9D_5$	14	C60H103O8	951.7653	291.2324/ 305.2481
$S^9M_5{}^9D_5$	15	C60H105O8	953.7810	291.2324/ 305.2481
$P^9M_5{}^{11}D_5$	16	C60H105O8	953.7810	291.2324/ 333.2794
$Ep^9M_5{}^9M_5$	17	C61H97O8	957.7184	291.2324
$Ln^9D_5{}^9D_5$	18	$C_{61}H_{101}O_8$	961.7497	305.2481
$L^9D_5{}^9D_5$	19	$C_{61}H_{103}O_8$	963.7653	305.2481
$Pn^9D_5{}^{11}D_5$	20	$C_{61}H_{105}O_8$	965.7810	305.2481/ 333.2794
$Pn^9D_5{}^{11}D_5$	21	$C_{61}H_{105}O_8$	965.7810	305.2481/ 333.2794
$O^9D_5{}^9D_5$	22	$C_{61}H_{105}O_8$	965.7810	305.2481
$P^9D_5{}^{11}D_5$	23	$C_{61}H_{107}O_8$	967.7966	305.2481/ 333.2794
$S^9D_5{}^9D_5$	24	$C_{61}H_{107}O_8$	967.7966	305.2481
$Ep^9M_5{}^9D_5$	25	C62H99O8	971.7340	291.2324/ 305.2481
$Ln^9M_5{}^{11}D_5$	26	C62H103O8	975.7653	291.2324/ 333.2794
$L^9M_5{}^{11}D_5$	27	C62H105O8	977.7810	291.2324/ 333.2794
$O^9M_5{}^{11}D_5$	28	C62H107O8	979.7966	291.2324/ 333.2794
$S^9M_5{}^{11}D_5$	29	C62H109O8	981.8123	291.2324/ 333.2794
$Dh^9M_5{}^9M_5$	30	C63H99O8	983.7340	291.2324
$Dp^9M_5{}^9M_5$	31	C63H101O8	985.7497	291.2324
$Ep^9D_5{}^9D_5$	32	C63H101O8	985.7497	305.2481
$Ln^9D_5{}^{11}D_5$	33	$C_{63}H_{105}O_8$	989.7801	305.2481/ 333.2794
$L^9D_5{}^{11}D_5$	34	$C_{63}H_{107}O_8$	991.7966	305.2481/ 333.2794
$O^{11}D_5{}^9D_5$	35	$C_{63}H_{109}O_8$	993.8123	305.2481/333.2794
$Pn^{11}D_5{}^{11}D_5$	36	$C_{63}H_{109}O_8$	993.8123	333.2794
$S^9D_5{}^{11}D_5$	37	$C_{63}H_{111}O_8$	995.8279	305.2481/ 333.2794

$Dh^9M_5{}^9D_5$	38	C64H101O8	997.7497	291.2324/ 305.2481
$P^{11}D_5{}^{11}D_5$	39	$C_{63}H_{113}O_8$	997.8436	333.2794
$Ep^9M_5{}^{11}D_5$	40	C64H103O8	999.7653	291.2324/ 333.2794
$Dp^9M_5{}^9D_5$	41	C64H103O8	999.7653	291.2324/ 305.2481
$Dh^9D_5{}^9D_5$	42	C65H103O8	1011.7653	305.2481
$Dp^9D_5{}^9D_5$	43	C65H105O8	1013.7810	305.2481
$Ep^9D_5{}^{11}D_5$	44	$C_{65}H_{105}O_8$	1013.7810	305.2481/ 333.2794
$Ln^{11}D_5{}^{11}D_5$	45	$C_{65}H_{109}O_8$	1017.8123	333.2794
$L^{11}D_5{}^{11}D_5$	46	$C_{65}H_{111}O_8$	1019.8279	333.2794
$O^{11}D_5{}^{11}D_5$	47	$C_{65}H_{113}O_8$	1021.8436	333.2794
$S^{11}D_5{}^{11}D_5$	48	$C_{65}H_{115}O_8$	1023.8592	333.2794
$Dh^9M_5{}^{11}D_5$	49	C66H105O8	1025.7810	291.2324/ 333.2794
$Dp^9M_5{}^{11}D_5$	50	C66H107O8	1027.7966	291.2324/ 333.2794
$Dh^9D_5{}^{11}D_5$	51	$C_{67}H_{107}O_8$	1039.7966	305.2481/ 333.2794
$Dp^9D_5{}^{11}D_5$	52	$C_{67}H_{109}O_8$	1041.8123	305.2481/ 333.2794
$Ep^{11}D_5{}^{11}D_5$	53	$C_{67}H_{109}O_8$	1041.8123	333.2794
$Dh^{11}D_5{}^{11}D_5$	54	$C_{69}H_{111}O_8$	1067.8279	333.2794
$Dp^{11}D_5{}^{11}D_5$	55	$C_{69}H_{113}O_8$	1069.8436	333.2794

* fatty acids are listed as follows: first conventional fatty acid listed followed by the two FuFAs in position two and three of the glycerol backbone with increasing molecular mass. This order must not reflect the correct order of fatty acids.

Table S5: Comparison of both chromatographic separation methods:

a) the old method:

Time [min]	Flow [mL/min]	Eluent A [%]	Eluent B [%]
0.00	0.300	65	35
5.00	0.300	50	50
12.00	0.300	0	100
18.00	0.300	0	100
20.00	0.300	50	50
29.00	0.300	50	50
30.00	0.300	50	50

Eluent A: methanol/ water (1:1, *v/v*) with 10 mM ammonium acetate; 0.1% formic acid

Eluent B: *iso*-propanol with 10 mM ammonium acetate; 0.1% formic acid

b) the new method:

Time [min]	Flow [mL/min]	Eluent A [%]	Eluent B [%]
0.00	0.325	55	45
7.00	0.325	40	60
28.00	0.325	18	82
29.00	0.325	1	99
34.90	0.325	1	99
35.00	0.325	55	45
40.00	0.325	55	45

Eluent A: acetonitrile/ water (6:4, *v/v*) with 5 mM ammonium formiate; 0.2% formic acid

Eluent B: *iso*-propanol/acetonitrile (9:1, *v/v*) with 5 mM ammonium formiate; 0.2% formic acid

Table S6: FuFA-containing TAGs in the fish oil I

TAG *	t_R [min]	molecular formula	$[M+H]^+$	fragment ions **	carbon-number	signal intensity (I) ***
$EpEp^9M_5$	17.94	$C_{62}H_{94}O_7$	951.7078	109.0652<br>165.1274<br>**291.2319**<br>643.4716<br>649.4824	59	2,820
$EpEp^{11}D_3$	18.46	$C_{63}H_{96}O_7$	965.7233	123.0808<br>151.1119<br>**305.2477**<br>643.4729<br>663.4989	60	626
$EpDh^9M_5$	18.60	$C_{64}H_{96}O_7$	977.7235	109.0652<br>165.1274<br>**291.2320**<br>649.4828<br>669.4878<br>675.4985	61	2,390
$EpDh^{11}D_3$	19.09	$C_{65}H_{98}O_7$	991.7382	123.0806<br>151.1118<br>**305.2476**<br>663.4982<br>669.4879<br>689.5142	62	393
$DhDh^9M_5$	19.21	$C_{66}H_{98}O_7$	1003.7407	109.0653<br>165.1276<br>**291.2321**<br>675.4998<br>695.5042	63	600
$DhDh^{11}D_3$	19.83	$C_{67}H_{100}O_7$	1017.7537	123.0806<br>151.1118<br>**305.2475**<br>689.5142<br>695.5032	64	492
ODh^9M_5	21.91	$C_{62}H_{100}O_7$	957.7580	109.0653<br>165.1275<br>**291.2320**<br>629.5154<br>649.5197<br>675.5015	59	342
$EpDp^{11}D_3$	19.96	$C_{65}H_{100}O_7$	993.7550	123.1171<br>151.1119<br>**305.2477**<br>663.4976<br>671.5035<br>691.5392	62	186
$EpEp^{11}D_5$	20.15	$C_{65}H_{100}O_7$	993.7537	123.0807<br>179.1430<br>**333.2790**<br>643.4721<br>691.5300	62	712
$EpDh^{11}D_5$	20.81	$C_{67}H_{102}O_7$	1019.7698	123.0806<br>179.1430<br>**333.2791**<br>669.4880<br>691.5297	64	12,000

				717.5453		
DhDh[11]D5	21.42	$C_{69}H_{104}O_7$	1045.7837	123.0807<br>179.1431<br>**333.2792**<br>695.5034<br>717.5453	66	4,030
EpDp[11]D5	21.48	$C_{67}H_{104}O_7$	1021.7852	123.0808<br>179.1432<br>**333.2789**<br>671.5043<br>691.5303<br>719.5609	64	285
DhDp[11]D5	22.12	$C_{69}H_{106}O_7$	1047.8010	123.0807<br>179.1431<br>**333.2791**<br>697.5198<br>717.5459<br>719.5611	66	2,140

* fatty acids are listed as follows: first two conventional fatty acids with the one with higher mass listed first, followed by the FuFA in position three of the glycerol backbone. This order must not reflect the correct order of fatty acids.

** from top to bottom: ion formed by McLafferty rearrangement, furan core ion, base peak ([FCO]$^+$) – formally [FuFA-OH]$^+$, printed in bold) and finally two or three [R-COOH]$^+$ ions

*** Signal intensity of the base peak, divided by a factor of 1000. In the case of FuFA-containing TAGs, this is the [FCO]$^+$ ion (formally [FuFA-OH]$^+$, formed by α-cleavage).

Table S7: FuFA-containing TAGs in the gilthead liver extract (fish oil II)

TAG *	t_R [min]	sum formula	[M+H]$^+$	fragment ions **	carbon-number	signal intensity (I) ***
PL^{9}D$_3$	18.30	$C_{55}H_{96}O_7$	869.7289	123.1170<br>151.1118<br>**277.2164**<br>575.5035<br>589.4826<br>613.4825	52	2,690
LO^{9}D$_3$	18.51	$C_{57}H_{98}O_7$	895.7355	123.1170<br>151.1118<br>**277.2164**<br>601.5191<br>613.4827<br>615.4984	54	3,920
OO^{7}D$_5$	19.49	$C_{57}H_{100}O_7$	897.7463	123.1170<br>179.1431<br>**277.2165**<br>603.5349<br>615.4988	54	884
PO^{7}D$_5$	19.79	$C_{55}H_{98}O_7$	871.6912	123.1170<br>179.1431<br>**277.2163**<br>577.5190<br>599.4825<br>615.4982	52	1,230
LO^{11}D$_5$	20.90	$C_{61}H_{106}O_7$	951.7839	123.1175<br>179.1436<br>**333.2790**<br>601.5187<br>669.5457<br>671.5647	58	29
OS^{7}D$_5$	21.28	$C_{57}H_{102}O_7$	899.7083	123.1170<br>179.1430<br>**277.2162**<br>605.5500<br>615.4982<br>617.5139	54	648
OS^{9}D$_3$	21.61	$C_{57}H_{102}O_7$	899.7087	123.1170<br>151.1118<br>**277.2164**<br>605.5502<br>615.4984<br>617.5148	54	1,030
LO^{9}M$_5$	23.27	$C_{58}H_{100}O_7$	909.7542	109.0652<br>165.1275<br>**291.2319**<br>601.5193<br>627.4999<br>629.5153	55	522
PO^{9}M$_5$	24.63	$C_{56}H_{100}O_7$	885.7556	109.0652<br>165.1275<br>**291.2319**<br>577.5192<br>603.4987<br>629.5141	53	938
OO^{9}M$_5$	24.71	$C_{58}H_{102}O_7$	911.7703	109.0653	55	

				165.1275<br>**291.2320**<br>603.5353<br>629.5158		245

* fatty acids are listed as follows: first two conventional fatty acids with the one with higher mass listed first, followed by the FuFA in position three of the glycerol backbone. This order must not reflect the correct order of fatty acids.

** from top to bottom: ion formed by McLafferty rearrangement, furan core ion, base peak ($[FCO]^+$) – formally $[FuFA\text{-}OH]^+$, printed in bold) and finally two or three $[R\text{-}COOH]^+$ ions

*** Signal intensity of the base peak, divided by a factor of 1000. In the case of FuFA-containing TAGs, this is the $[FCO]^+$ ion (formally $[FuFA\text{-}OH]^+$, formed by α-cleavage).

Table S8: Replicate LC-orbitrap-MSMS runs (n=1) of a gilthead liver extract (fish oil II) with relative abundances four conventional TAGs (relative to LLL = 100%) and five FuFA-containing TAGs (relative to LO^9D_3 = 100%).

TAG	run 1 [%]	run 2 [%]	run 3 [%]	mean	STDEV (%)
LnLnLn	31.3	41.8	35.6	36.2	5.3 (14.6%)
POL	1.2	1.0	1.0	1.1	0.1 (9.1%)
LLL	100.0 *	100.0 *	100.0 *	100	100
OOO	14.9	11.7	4.5	10.4	5.3 (51%)
PL^9D_3	55.3	81.9	117.7	85.0	31.3 (36.8%)
LO^9D_3	100.0 *	100.0 *	100.0 *	100	100
OO^7D_5	58.9	51.6	96.7	69.1	24.3 (35.1%)
PO^7D_5	26.6	45.1	33.1	34.9	9.4 (26.9)
OS^9D_3	24.7	24.5	19.8	23.0	2.8 (12.2%)

* LO^9D_3 as the FuFA-containing TAG with the highest intensity was set to 100%, except run 3, where PL^9D_3 shows the highest intensity.

Table S9: Estimated content of 9D5 in the FuFA-containing TAGs of the king oyster mushroom.

	**Intensity I of the**	**Contribution of 9D5 to the sample [mg/100 g dry weight]**
XY^9D_5****	570	0.1
$15{:}0\text{-}Ln^9D_5$	1,010	0.2
$L^9D_5{}^9D_5$	9,980	2.4
PnL^9D_5	2,110	0.5
LL^9D_5	66,800	16.0
$15{:}0\text{-}L^9D_5$	3,770	0.9
$O^9D_5{}^9D_5$	1,430	0.3
LO^9D_5	29,100	7.0
$15{:}0\text{-}O^9D_5$	1,280	0.3
$17{:}0\text{-}L^9D_5$	820	0.2
PO^9D_5	9,550	2.3
OO^9D_5	10,100	2.4
OS^9D_5	980	0.2
**sum**	**137,490**	**33**

Table S10: Estimated content of 9M5, 11D3 and 11D5 in the FuFA-containing TAGs of the fish oil I.

	**Intensity I**	**FuFA [mg/ 100 g lipids]**
EpEp$^{9}M_{5}$	2,820	120
EpDh$^{9}M_{5}$	2,390	110
DhDh$^{9}M_{5}$	600	26
ODh$^{9}M_{5}$	342	15
**sum**	**6,152**	**270**
EpEp$^{11}D_{3}$	626	170
EpDh$^{11}D_{3}$	393	104
DhDh$^{11}D_{3}$	492	130
EpDp$^{11}D_{3}$	186	49
**sum**	**1,697**	**450**
EpEp$^{11}D_{5}$	712	12
EpDh$^{11}D_{5}$	12,000	194
DhDh$^{11}D_{5}$	4,030	65
EpDp$^{11}D_{5}$	285	4.6
DhDp$^{11}D_{5}$	2,140	35
**sum**	**19,167**	**310**

Figures

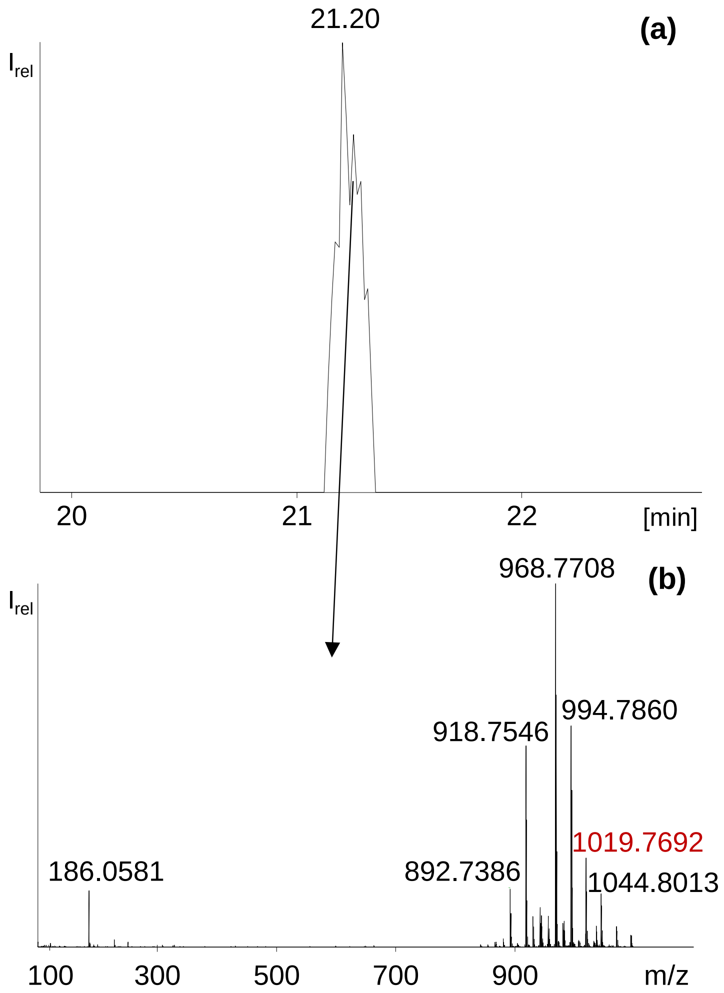

Fig. S1: (**a**) LC-Orbitrap-HRMS chromatogram of fish oil I with the extracted $[M+H]^+$ ion *m/z* 1019.7704 in MS^1 with 4 ppm. (**b**) MS^1 mass spectrum at retention time 21.25 min.
